# Structural Inheritance and Redox Performance of Nanoporous Electrodes from Nanocrystalline Fe_85.2_B_10-14_P_0-4_Cu_0.8_ Alloys

**DOI:** 10.3390/nano7060141

**Published:** 2017-06-08

**Authors:** Chaoqun Fu, Lijun Xu, Zhenhua Dan, Akihiro Makino, Nobuyoshi Hara, Fengxiang Qin, Hui Chang

**Affiliations:** 1 Tech Institute for Advanced Materials, College of Materials Science and Engineering, Nanjing Tech University, Nanjing 210009, China; 840350217@njtech.edu.cn (C.F.); xulijun@njtech.edu.cn (L.X.); ch2006@njtech.edu.cn (H.C.); 2 The Synergetic Innovation Center for Advanced Materials, Nanjing Tech University, Nanjing 210009, China; 3 Institute for Materials Research, Tohoku University, Sendai 9808577, Japan; amakino@imr.tohoku.ac.jp; 4 Department of Materials Science, Tohoku University, Sendai 9808579, Japan; haran@material.tohoku.ac.jp; 5 School of Materials Science and Engineering, Nanjing University of Science and Technology, Nanjing 210094, China; fengxiangqin@njust.edu.cn

**Keywords:** nanoporous material, amorphous materials, nanocrystalline soft magnetic material, corrosion, dealloying

## Abstract

Nanoporous electrodes have been fabricated by selectively dissolving the less noble α-Fe crystalline phase from nanocrystalline Fe_85.2_B_14–*x*_P*_x_*Cu_0.8_ alloys (*x*= 0, 2, 4 at.%). The preferential dissolution is triggered by the weaker electrochemical stability of α-Fe nanocrystals than amorphous phase. The final nanoporous structure is mainly composed of amorphous residual phase and minor undissolved α-Fe crystals and can be predicted from initial microstructure of nanocrystalline precursor alloys. The structural inheritance is proved by the similarity of the size and outlines between nanopores formed after dealloying in 0.1 M H_2_SO_4_ and α-Fe nanocrystals precipitated after annealing of amorphous Fe_85.2_B_14−*x*_P*_x_*Cu_0.8_ (*x* = 0, 2, 4 at.%) alloys. The Redox peak current density of the nanoporous electrodes obtained from nanocrystalline Fe_85.2_B_10_P_4_Cu_0.8_ alloys is more than one order higher than those of Fe plate electrode and its counterpart nanocrystalline alloys due to the large surface area and nearly-amorphous nature of ligaments.

## 1. Introduction

Dealloying, as a process of selective dissolution of less noble elements in alloys, has been extensively used to prepare nanoporous (np) metals, including np Pt, np Pd, np Au, np Ag and np Cu, in recent decades [1,2]. The large surface area of these nanoporous metals enhances their catalytic performance, as well as their surface Raman scattering effect and their cost effectiveness [3,4,5]. The dealloying method has mainly been focused on binary or ternary alloys which consist of elements with different electrode potentials. A difference of more than a few hundredth milli-volts in the electrode potential is regarded as a prerequisite for motivating the selective dissolution of less noble elements [6]. The dealloying strategy has been extended to the fabrication of porous metallic glasses via the selective dissolution of less noble phase in two dual phase amorphous alloys: Y_20_Ti_36_Al_24_Co_20_ [7] and La_27.5_Zr_27.5_Al_25_Cu_10_Ni_10_ [8]. While these porous metallic glasses with different architectures were successfully fabricated using dealloying techniques, the fabrication technique results in a minimum pore size restricted to the micro-scale. It should be noted that a much smaller pore size (e.g., <100 nm) is required in order to achieve the high specific surface area associated with enhanced performance. In nanocrystalline alloys consisting of two phases, i.e., Ti_50_Cu_50_ [9], Ti_59_Al_41_ [10], Zr_2_Ni alloy [11], Ni-based superalloys [12], the preferential dissolution behavior of less noble Ti_2_Cu, α_2_-Ti_3_Al,Zr and γ′-Ni_3_Al is more pronounced than that of the γ-TiCu, γ-TiAl, Zr_2_Ni and γ-Ni phases during the formation of the nanoporous structure. The final porous structure reflects the characteristics of the initial microstructure of the crystalline precursor alloys [9,11,12]. The unique hetero-amorphous microstructure of Fe-based nanocrystalline soft magnetic alloys is composed of α-Fe nanocrystals and a surrounding amorphous phase, and provides excellent soft magnetic properties, such as low coercivity, high saturated magnetic flux density, and low core loss, etc. [13,14,15]. The specific surface area of the porous structure obtained from Cu powder embedded layered Cu_47_Ti_33_Zr_11_Ni_8_Si_1_ bulk metallic glasses after leaching the Cu phase in HNO_3_ has been reported to be 23.5 m^2^/g [16]. The selective dissolution of Cu powder embedded in Cu_47_Ti_33_Zr_11_Ni_8_Si_1_ bulk metallic glasses suggests that the electrochemical stability of the crystalline Cu phase is weaker and that the amorphous phase remains after dealloying. The dissolution behavior of α-Fe nanocrystals in the amorphous matrix of Fe-based nanocrystalline alloys remains unclear although the coupling behavior of these alloys is similar to that of Cu embedded metallic glass. Unlike the other alloys mentioned above, it is possible to fabricate a nanoporous structure from heterogeneous Fe-based nanocrystalline alloys. It is worth noting that the surface area of a porous structure is more or less constant when the pore size is above 1 μm and is independent of the volume fraction of pores. Once the pore size is reduced to below 100 nm, the surface area drastically increases and becomes sensitive to the volume fraction of the pores [16]. Since the particle size of the nanocrystals in nanocrystalline Fe-based soft magnetic alloys has been reported to be in the range of a few tenths of nanometers [13,14,15], the surface area of the nano-sized porous architecture obtained from nanocrystalline precursor alloys is highly anticipated to be considerably larger than that of dual phase metallic glass precursors.

Nanocrystalline Fe-based soft magnetic alloys (i.e., Fe_83.3_Si_4_B_8_P_4_Cu_0.7_, Fe_85-86_Si_1-2_B_8_P_4_Cu_1_ alloys) have been developed last decade with saturated magnetic flux density of higher than 1.8 T and coercivity of less than 10 A m^−1^ in comparison to amorphous Fe-Si-B ribbons and Fe-Si magnetic steels [13,14,15]. The addition of P and Cu elements into Fe-Si-B alloys can effectively improve the soft magnetic performance through increasing the nucleation density and suppressing the growth of the α-Fe nanocrystals during the flash annealing [13,15,17,18]. Beside the corrosion properties and the glass forming ability of Fe-Si-B alloys are enhanced by the addition of P element, the addition of P is helpful to reduce the size of grains [19,20]. Therefore, the new serials of the Fe_85.2_B_14−*x*_P*_x_*Cu_0.8_ alloys (*x* = 0, 2, 4 at.%) is designed to investigate the effect of P addition on the refinement of the α-Fe nanocrystals and then the effect of the refined α-Fe nanocrystals on the characteristics of the final nanoporous structure. As mentioned above, an alternative strategy for the formation of a porous structure with a characteristic pore size of few tenths of nanometers is proposed in the present research. Nanoporous structure with an ultra-large surface area, which is helpful to better the catalytic properties [21] and enhance the reduction and oxidization reactions (Redox) in the metal-air battery systems [22], might obtain after the selective dissolution of α-Fe nanocrystals in nanocrystallineFe_85.2_B_14−*x*_P*_x_*Cu_0.8_ alloys (*x* = 0, 2, 4 at.%). For instance, the negative electrodes in an alkaline condition undergoes the oxidation and reduction reaction during discharging and charging. Therefore, the efficiency of the Redox reaction is able to be improved by introducing the nanoporous structure into Fe-air batteries [23,24].

Nanocrystalline Fe_85.2_B_14−*x*_P*_x_*Cu_0.8_ alloys (*x* = 0, 2, 4 at.%) were immersed in 0.1 M H_2_SO_4_ solution to fabricate the porous architecture. The main focus of this study was to determine the dependence of the final nanoporous structure (pore size distribution and surface area) on the initial nanocrystalline properties (alloy composition and microstructure) of Fe-based precursor alloys. These nanoporous electrodes are expected to be mainly composed of the residual amorphous phases. Their properties of reduction and oxidization reactions (Redox) in an alkaline condition is evaluated in comparison to pure Fe bulk electrode.

## 2. Materials and Experimental Procedure

Fe_85.2_B_14−*x*_P*_x_*Cu_0.8_ (*x* = 0, 2, 4 at.%) ribbons with a width of 10 mm and thickness of about 18 μm were fabricated by the induction melting and single roller melt spinning technique. The details has been illustrated in previous papers [13]. The linear velocity of the single-roller melt spinning was set at 42 m s^−1^. Thermal property of as-spun alloys was evaluated with a differential scanning calorimeter (DSC) at a heating rate of 40 K min^−1^ under an Ar gas flow. Heat treatment of as-spun alloys was conducted at the onset crystallization temperature of three alloys for 600 s under an Ar flow. Crystalline states were identified by using an X-ray diffractometor (XRD, Rigaku, SmartLab, Rigaku Co., Tokyo, Japan) with a CuK_α_ radiation. The scan speed was set at 1° min^−1^.

The potentiodynamic polarization curves were measured in 0.1 M H_2_SO_4_ solution with a scan rate of 50 mV min^−1^. The dealloying was conducted in 0.1 M H_2_SO_4_ (AR, Sinopharm Chemical Reagent Co., Ltd, Shanghai, China) solution for 7.2 ks. The cyclic voltammograms (CV) of nanocrystalline and nanoporous Fe-B-P-Cu alloys were obtained in 6.0 M KOH (AR, Sinopharm Chemical Reagent Co., Ltd, Shanghai, China) solution with the scan rate was 50 mV s^−1^. All the potential was referred to the Ag/AgCl (3.33 M KCl) electrode unless otherwise stated. The microstructure was characterized by a scanning electron microscope (SEM, JEOL 4610, JEOL Ltd., Tokyo, Japan) and a transmission electron microscope (TEM, JEOL, JEM-HC2100 and JEOL-ARM210F, JEOL Ltd., Tokyo, Japan), and the samples for TEM observation were prepared by focused ion beam (FIB) method. The size of α-Fe grains and nanopores was estimated by XRD analysis using Scherrer’s equation, SEM and TEM observation. The average size of α-Fe nanocrystals and nanopores was estimated statistically over 125 particles from more than three SEM and TEM images for each condition by using single chord length method with Nanomeasure^®^ software.

## 3. Results and Discussion

### 3.1. Structural Characteristics of Amorphous and Nanocrystalline Fe_85.2_B_14−x_P_x_Cu_0.8_ Precursor Alloys

**Figure 1:**
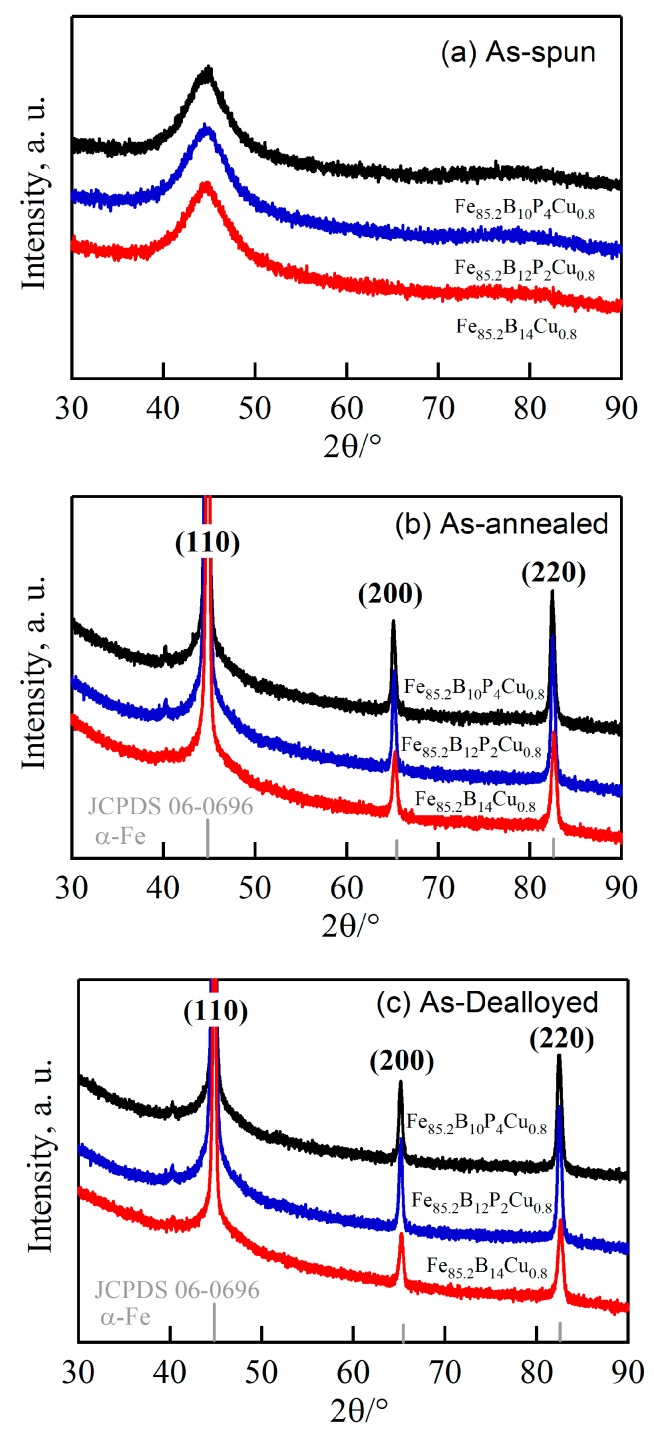
X-ray diffractometor (XRD) patterns of as-quenched amorphous (**a**) as-annealed nanocrystalline (**b**) and as-dealloyed nanoporous (**c**) Fe_85.2_B_14−*x*_P_*x*_Cu_0.8_ alloys (*x* = 0, 2, 4 at.%) alloys.

The XRD patterns of the as-spun Fe_85.2_B_14−*x*_P*_x_*Cu_0.8_ (*x* = 0, 2, 4 at.%) alloys in Figure 1a consisted of a single halo diffraction peak at 2Θ = 44°, indicating that the as-spun ribbons were amorphous. The onset crystallization temperatures, *T*_x1_, and secondary crystallization temperatures, *T*_x2_, of the as-spun ribbons were confirmed to be 662 and 772 K for amorphous Fe_85.2_B_14_Cu_0.8_ alloy, 680 and 798 K for amorphous Fe_85.2_B_12_P_2_Cu_0.8_ alloy, 680 and 801 K for amorphous Fe_85.2_B_10_P_4_Cu_0.8_ alloy, respectively. *T*_x1_ and *T*_x2_ increased with increasing P content. The increasing difference, Δ*T* = *T*_x2_₋*T*_x1_, in the crystallization temperatures with increasing P content indicates that the thermal stability of precipitated phase became higher. 

**Figure 2:**
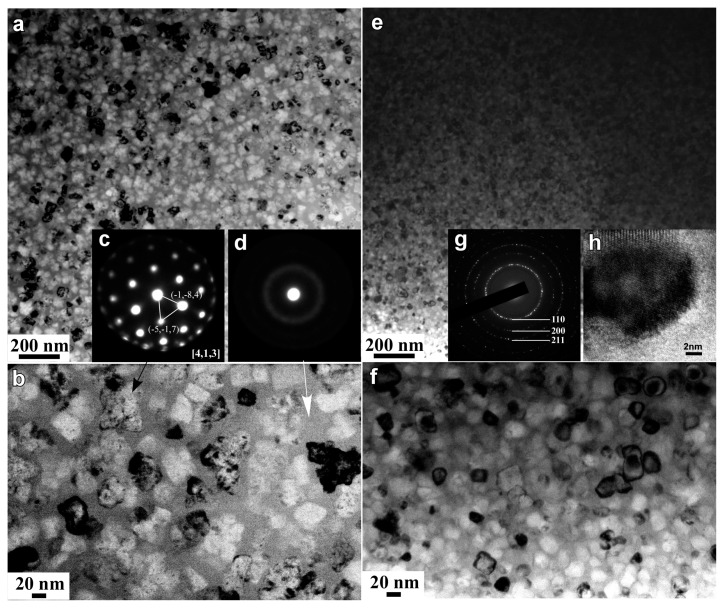
Bright field TEM image (**a**,**e**) and magnified bright field TEM image (**b**,**f**) of nanocrystalline Fe_85.2_B_14_Cu_0.8_ and Fe_85.2_B_10_P_4_Cu_0.8_ alloys. Selected area nano-diffraction patterns (**c**,**d**) at the zone which the arrows marked in nanocrystalline Fe_85.2_B_14_Cu_0.8_ alloy. The selected area diffraction pattern (**g**) and high-resolution TEM image (**h**) of nanocrystalline Fe_85.2_B_10_P_4_Cu_0.8_ alloy.

**Figure 3:**
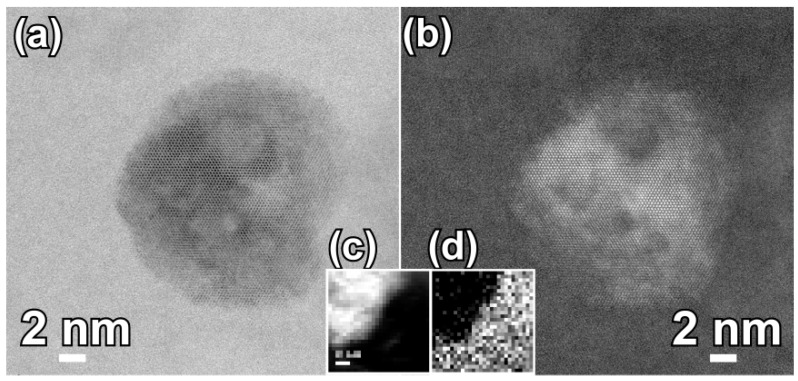
High-resolution bright field transmission electron microscope (TEM) image (**a**) and high angular dark field TEM image (**b**) of Fe_85.2_B_10_P_4_Cu_0.8_ alloy after annealing at 680 K for 600 s. EELS image (**c**) and distribution of B element (**d**) at the interface of α-Fe grain and residual amorphous matrix.

Three diffraction peaks at 2Θ of about 44°, 65° and 82° in the XRD patterns of the annealed alloys in Figure 1b are assigned to α-Fe (110), (200) and (220), indicating that the α-Fe phase was precipitated after annealing. The particle sizes of α-Fe nanocrystals, *D*_1_, were calculated to be 32 nm for annealed Fe_85.2_B_14_Cu_0.8_ ribbon, 28 nm for annealed Fe_85.2_B_12_P_2_Cu_0.8_ ribbon, and 25 nm for annealed Fe_85.2_B_10_P_4_Cu_0.8_ ribbon, respectively, using the Scherrer equation (*D* = *Kλ*/*β*sin*θ*) [25]. As shown in Figure 2a,b, two phases coexisted in the nanocrystalline Fe_85.2_B_14_Cu_0.8_ matrix: irregularly-shaped nanocrystals and continuously distributed phase. As indicated by the selected nano-sized area diffraction patterns (Figure 2c) at the regions which arrows marked in Figure 2b, the irregularly-shaped nanocrystals were mainly composed of α-Fe and in a crystalline state. On the other hand, the typical single halo diffraction ring in Figure 2d corresponding to the residual phase arrow marked demonstrates that the structure of the continuous phase was amorphous [26]. The diffraction patterns in Figure 2c,d are an evidence of the coexistence of the dual phases of α-Fe nanocrystals and residual amorphous phase. The grain size of α-Fe nanocrystals, *D*_2_, was confirmed to be 31 nm for annealed Fe_85.2_B_14_Cu_0.8_ ribbon, 25 nm for annealed Fe_85.2_B_12_P_2_Cu_0.8_ ribbon, and 18 nm for annealed Fe_85.2_B_10_P_4_Cu_0.8_ ribbon by TEM analysis, respectively. Compared with those of as-annealed Fe_85.2_B_14_Cu_0.8_ alloys, the grain size of as-annealed Fe_85.2_B_10_P_4_Cu_0.8_ alloy became slightly smaller because of the P alloying. As shown in Figure 2e,f, the shape of the α-Fe nanocrystals in the as-annealed Fe_85.2_B_10_P_4_Cu_0.8_ ribbon was almost cubic. The selected area diffraction pattern (SADP) over a wider area in Figure 2g, is typical for the α-Fe phase [13,14,15]. The high-resolution TEM image in Figure 2h shows the characteristics of the nanocrystals and the interplanar spacing is 0.16 nm which is matched with *bcc* α-Fe (111). These observations suggest that the particle size of α-Fe nanocrystals was reduced by the addition of P to the Fe_85.2_B_14_Cu_0.8_ alloy. The elemental distribution profiles of Cu, Fe and P elements analyzed by TEM-EDX elemental mapping are shown in Figure S1 (the Supplementary Files). The P element distributes in the amorphous phase with a slightly higher concentration in comparison to that in α-Fe nanocrystals. This fact is consistence with those in the published papers [13,15,17,18]. Furthermore, the high-resolution TEM image in Figure 3a shows the clear crystalline state of single α-Fe nanocrystal indicating by the regular pattern in the middle region and amorphous nature of the surrounding residue. The darker color of the crystal indicates that the weight element, mainly Fe, enriches. The high angular dark field TEM image shows the similar distribution with an opposite color in Figure 3b. The electron energy loss spectroscopy (EELS) analysis and the distribution profiles of B element at the interface region between α-Fe nanocrystal and adjacent amorphous residue are shown in Figure 3c,d. The dark region in the up-left corner indicates the lower distribution of B element of about 2.5 at.% in the α-Fe nanocrystal.

### 3.2. Structural Characteristics of Nanoporous Alloys

**Figure 4:**
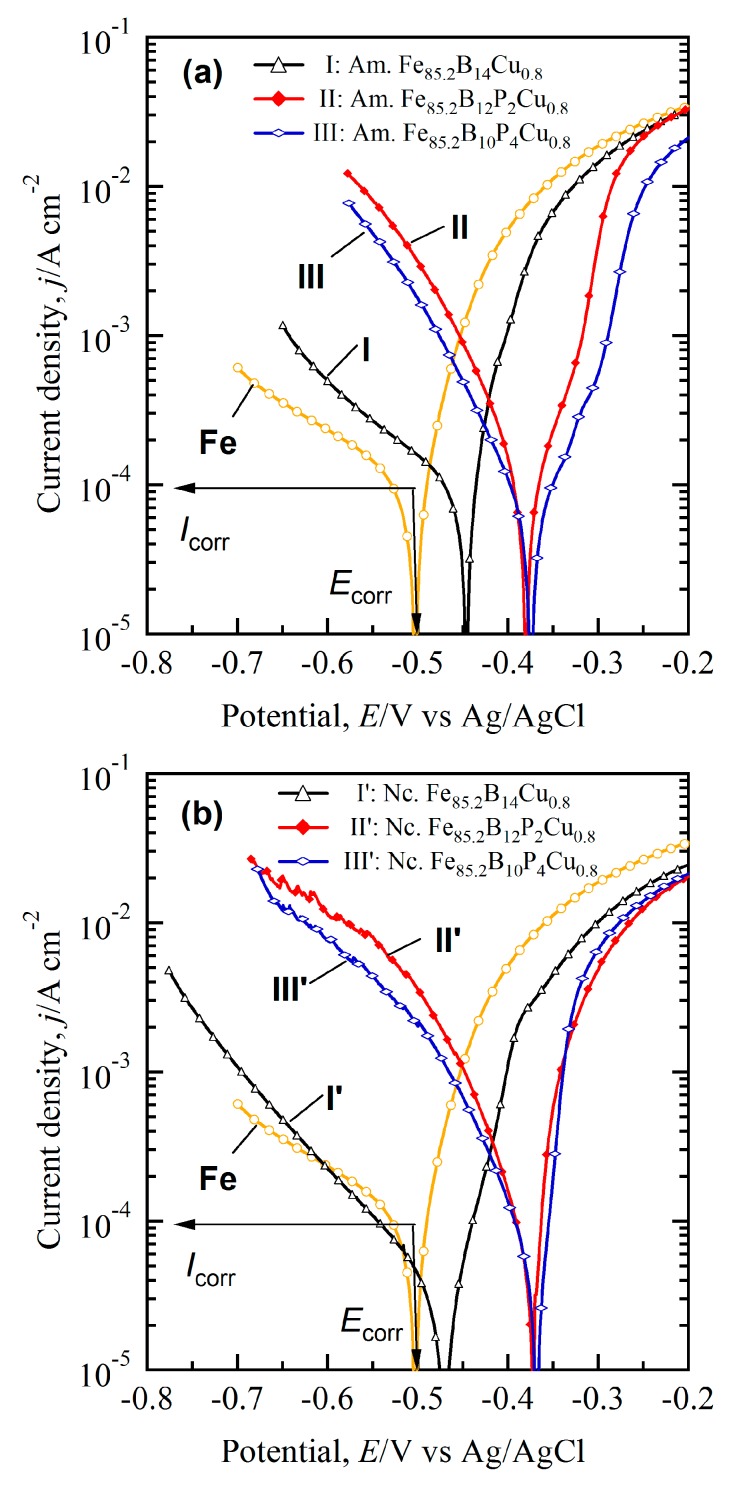
Potentiodynamic polarization curves of pure Fe, as-spun amorphous (**a**) and as-annealed nanocrystalline; (**b**) of Fe_85.2_B_14_Cu_0.8_ (I, I′), Fe_85.2_B_12_P_2_Cu_0.8_ (II, II′) and Fe_85.2_B_10_P_4_Cu_0.8_ (III, III′) ribbons in 0.1 M H_2_SO_4_ solution, and Tafel slope analysis of the polarization curves of Fe plate are inserted. Am.: Amorphous; Nc.: Nanocrystalline.

Figure 4 shows the anodic polarization behaviors of pure Fe, and amorphous and nanocrystalline alloys in 0.1 M H_2_SO_4_ solution. The logarithm plot of Fe with a purity of 99.999 mass%, which was used as a reference material, is also shown in Figure 4. The corrosion current, *I*_corr_, and corrosion potential, *E*_corr_, were determined by the Tafel slope method [27]. On basis of the Tafel analysis, the corrosion potential (*E*_corr_) of pure iron plate are −0.5 V. The logarithm plots of the anodic polarization curves in Figure 4a,b clearly demonstrate the different electrochemical behavior of the amorphous and nanocrystalline alloys (I and I′: Fe_85.2_B_14_Cu_0.8_; II and II′: Fe_85.2_B_12_P_2_Cu_0.8_; III and III′: Fe_85.2_B_10_P_4_Cu_0.8_). The values of *E*_corr_ for nanocrystalline Fe_85.2_B_14_Cu_0.8_, Fe_85.2_B_12_P_2_Cu_0.8_ and Fe_85.2_B_10_P_4_Cu_0.8_ alloys were confirmed to be −0.47 V, −0.37 V, and −0.37 V. Those of amorphous counterparts were −0.45 V, −0.38 V and −0.37 V, respectively. The absence of passivation regions in the anodic polarization curves indicates that all alloys were in an active dissolution state. As explained by Pickering in his work on binary model alloys [28], the critical potential (*E*_c_) can be determined by subjecting the alloys to anodic polarization. The values of *E*_c_ for alloys tested in an active dissolution state are considered to be close to their *E*_corr_ in the present condition. Compared with pure Fe electrode, values of *E*_corr_ for the amorphous counterpart alloys are shifted to right and the amorphous and nanocrystalline counterpart alloys have the better electrochemical stability than the α-Fe nanocrystals. A larger difference in *E*_corr_ was noted with higher P content. The *I*_corr_ of amorphous alloys was slightly smaller than that of pure Fe. It should be noted that the chemical composition of the α-Fe nanocrystals was 96 at.% Fe, 1.5 at.% P and 2.5 at.% B in the case of the nanocrystalline Fe_85.2_B_10_P_4_Cu_0.8_ alloy. On the other hand, the B and P content in the amorphous region adjacent to α-Fe nanocrystals was higher than that in the α-Fe nanocrystals, as shown in Figure S1 (Supplementary file). Since the high content of Fe in α-Fe nanocrystals (about 96 at.%), pure Fe plate is used to simulate the polarization behavior of α-Fe nanocrystals. When pure Fe and these amorphous alloys are coupled, pure Fe serves as anodes and these amorphous alloys function as cathodes. As a result, pure Fe undergoes the anodic dissolution and galvanic dissolution is motivated by the difference in *E*_corr_. In the nanocrystalline matrix, the α-Fe nanocrystals are considered to have the similar electrochemical behavior in 0.1 M H_2_SO_4_ solution. The α-Fe nanocrystals with lower *E*_corr_ serve as anodes and start the preferential dissolution in the form of micro-coupling cells due to the difference in *E*_corr_ between α-Fe nanocrystals and surrounding amorphous phase. Galvanic corrosion thus occurs on α-Fe nanocrystals and might lead to the formation of the pores.

**Figure 5:**
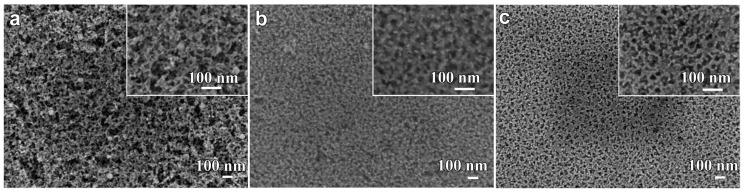
SEM morphology of nanocrystalline Fe_85.2_B_14_Cu_0.8_ (**a**) Fe_85.2_B_12_P_2_Cu_0.8_; (**b**) and Fe_85.2_B_10_P_4_Cu_0.8_; (**c**) ribbons after immersion in 0.1 M H_2_SO_4_ solution for 7.2 ks.

**Figure 6:**
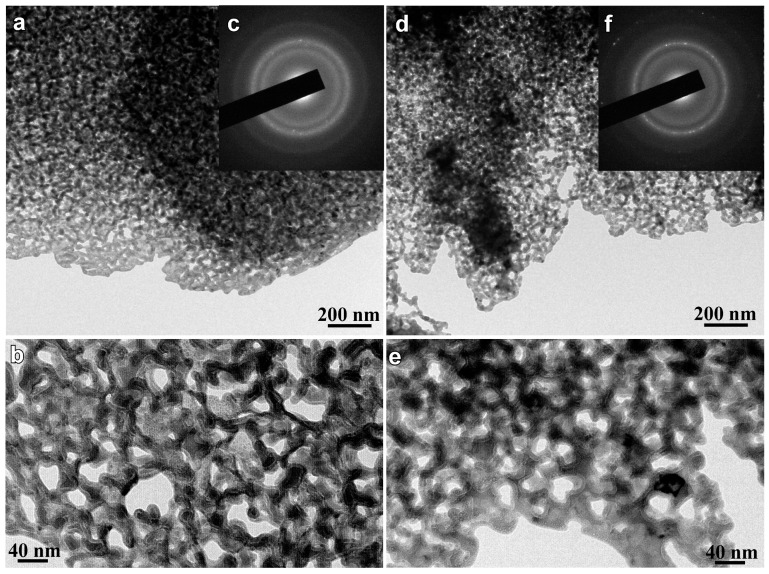
Bright field TEM image (**a**,**d**), magnified bright field TEM image (**b**,**e**) and selected area diffraction patterns (**c**,**f**) of nanocrystalline Fe_85.2_B_14_Cu_0.8_ and Fe_85.2_B_10_P_4_Cu_0.8_ alloys after free immersion in 0.1 M H_2_SO_4_ solution for 7.2 ks.

Selective dissolution of as-annealed Fe-B-P-Cu alloys in 0.1 M H_2_SO_4_ solution for 7.2 ks under a free corrosion condition causes the formation of the unique surface morphology in Figure 5. The porous structure formed on the nanocrystalline Fe_85.2_B_14_Cu_0.8_ alloy is sponge-like in structure with relatively large pores and narrow ligaments. A porous structure with a pore size of less than 100 nm was obtained for all the nanocrystalline alloys. The pore size of these porous structures was confirmed to be 25 nm for Fe_85.2_B_14_Cu_0.8_ alloy, 21 nm for Fe_85.2_B_12_P_2_Cu_0.8_ alloy and 16 nm for Fe_85.2_B_10_P_4_Cu_0.8_ alloy, respectively. Smaller nanopores were obtained in nanoporous structures from precursor alloys with a higher P content. The TEM images in Figure 6a,b,d,e show the inner nanoporous structures obtained from nanocrystalline Fe_85.2_B_14_Cu_0.8_ and Fe_85.2_B_10_P_4_Cu_0.8_. Although some irregularly-shaped nanopores with sizes larger than 50 nm were formed, the average pore size was confirmed to be 19 nm. The nanoporous structure formed on nanocrystalline Fe_85.2_B_10_P_4_Cu_0.8_ alloy shown in Figure 6d was more uniformly shaped and had an average pore size of 12 nm. A similar SADP for dealloyed Fe_85.2_B_14_Cu_0.8_ alloy was obtained from the residual phase of dealloyed nanocrystalline Fe_85.2_B_10_P_4_Cu_0.8_. The presence of the halo rings in the SADP of the residual phase in Figure 6c,f indicates that the residue was mainly consisted of amorphous phase. With the exception of those from the amorphous residual Fe-B-P-Cu phase, the diffraction rings can be attributed to minor crystalline undissolved α-Fe crystals which can be detected in the black particles in Figure 6e. The XRD patterns of as-dealloyed alloys in Figure 1c also demonstrates that the α-Fe nanocrystals are not fully dissolved after dealloying.

### 3.3. Structural Inheritance of Nanoporousalloys 

**Figure 7:**
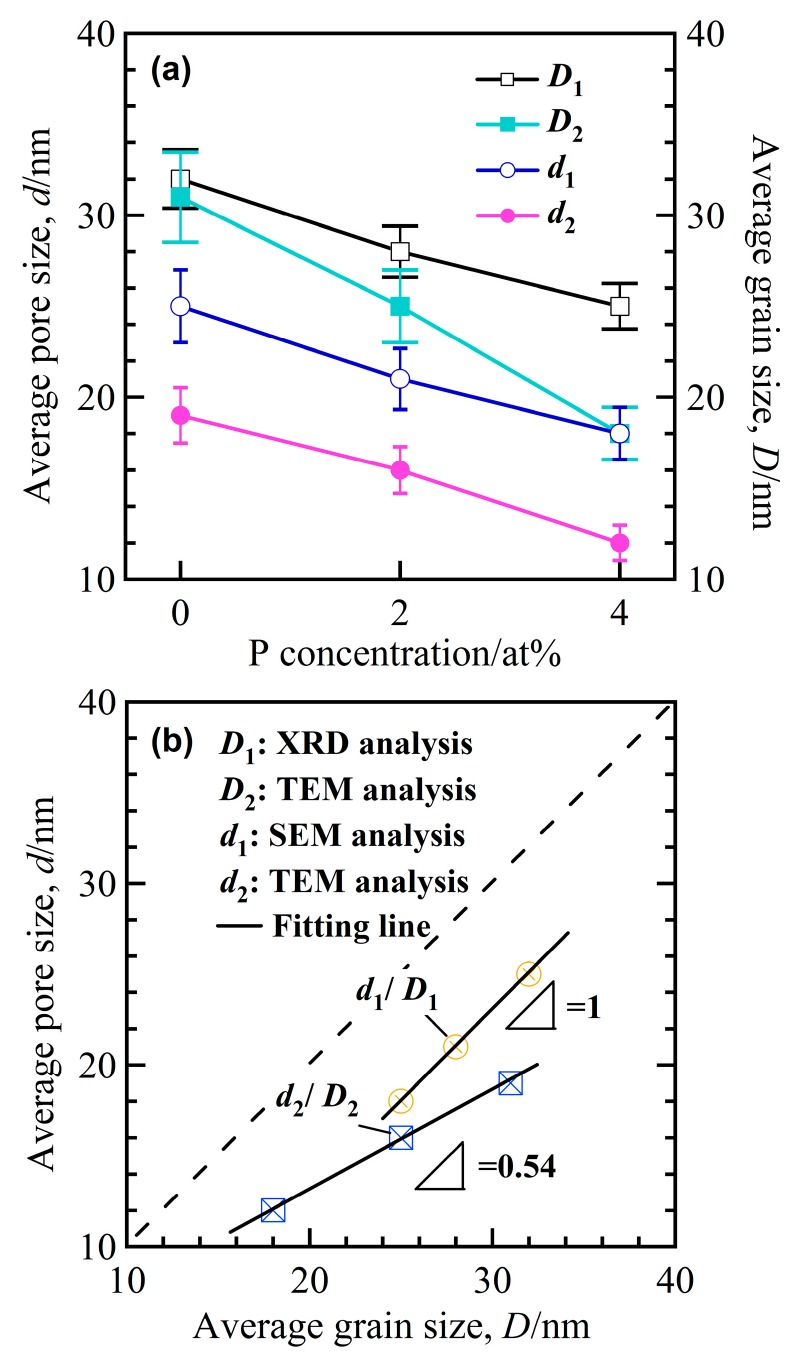
The change dependency of grain size, *D* (*D*_1_ from XRD analysis; *D*_2_ from TEM analysis), and pore size, *d* (*d*_1_ from SEM analysis; *d*_2_ from TEM analysis), on the P concentration (**a**) and the linear dependency of *d*_1_/*D*_1_ and *d*_2_/*D*_2_ (**b**).

As demonstrated in Figure 5 and Figure 6, the similarity of the shape and size between the α-Fe nanocrystals and nanopores, the structural inheritance between the nanocrystalline precursor alloys and nanoporous alloys can be outlined. The statistical data of grain size and pore size are presented in Figure S2 (Supplementary file), and the larger divergences of the distribution of the grain size and nanopore size of Fe_85.2_B_14_Cu_0.8_ alloys are considered to be due to the existence of the irregular α-Fe nanocrystals. On the other hand, the structural inheritance between nanocrystalline precursor alloys and nanoporous alloys can be reflected from the similarity of the size. The size comparison between the α-Fe grains and nanopores is shown in Figure 7a. The grain sizes were reduced with the increase of the P concentration of nanocrystalline Fe-B-P-Cu alloys, and the decrease of the nanopore size had almost the same change tendency. The slope of the fitting lines of *d*_1_/*D*_1_ and *d*_2_/*D*_2_ is confirmed to be 1 and 0.54, which indicates that the high reliability of the analysis method such as SEM can predict the final nanoporous structure more accurately. Here the new prediction methodology for the nanoporous structure of nanocrystalline Fe-based soft magnetic alloys can be proposed on the basis of the present results. The literature value of structural characteristics of the nanocrystalline precursor alloys can be used for the precise prediction of the final nanoporous structure. The present finding can explain that the large nanopores in Figure 6b formed after the selective dissolution of irregularly-shaped nanocrystals with a large size shown in Figure 2b. In fact, the unique nanoporous microstructure formed as a result of the selective dissolution of α-Fe nanocrystals under the interaction of micro-coupling cells between α-Fe nanocrystals and residual amorphous phases [29]. The difference of *E*_corr_ between Fe plate and the amorphous alloys in Figure 4 proves the selective dissolution of α-Fe nanocrystals in H_2_SO_4_ solution. The nearly-amorphous nanoporous alloy can be fabricated by the proposed method.

### 3.4. Enhanced Redox Performance of Nanocrystalline and Nanoporousalloys 

**Figure 8:**
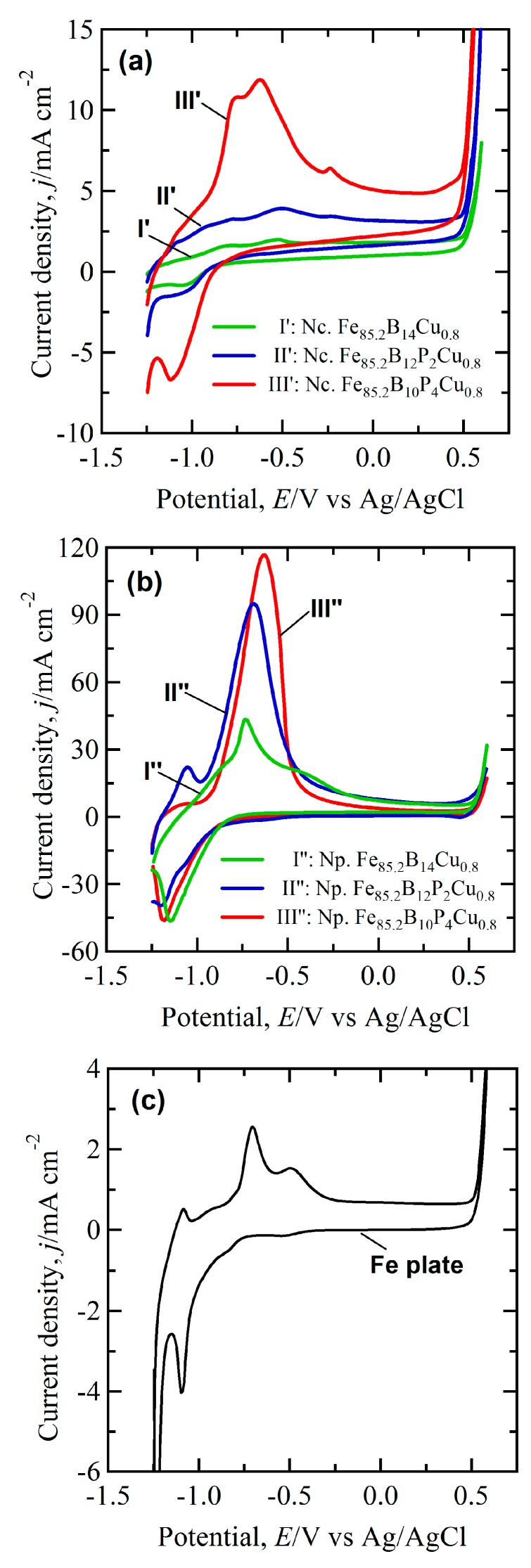
CV curves of nanocrystalline (**a**) nanoporous; (**b**) electrodes and pure Fe plate; (**c**) in 6.0 M KOH solution. Scan rate: 50 mV/s. Nanocrystalline electrodes: Fe_85.2_B_14_Cu_0.8_ (I′), Fe_85.2_B_12_P_2_Cu_0.8_ (II′) and Fe_85.2_B_10_P_4_Cu_0.8_ (III′) alloys after annealing of amorphous precursor alloys. Nanoporous electrodes: Fe_85.2_B_14_Cu_0.8_ (I′′), Fe_85.2_B_12_P_2_Cu_0.8_ (II′′) and Fe_85.2_B_10_P_4_Cu_0.8_ (III′′) alloys after dealloying of nanocrystalline precursor alloys in 0.1 M H_2_SO_4_ solution for 7.2 ks.

**Figure 9:**
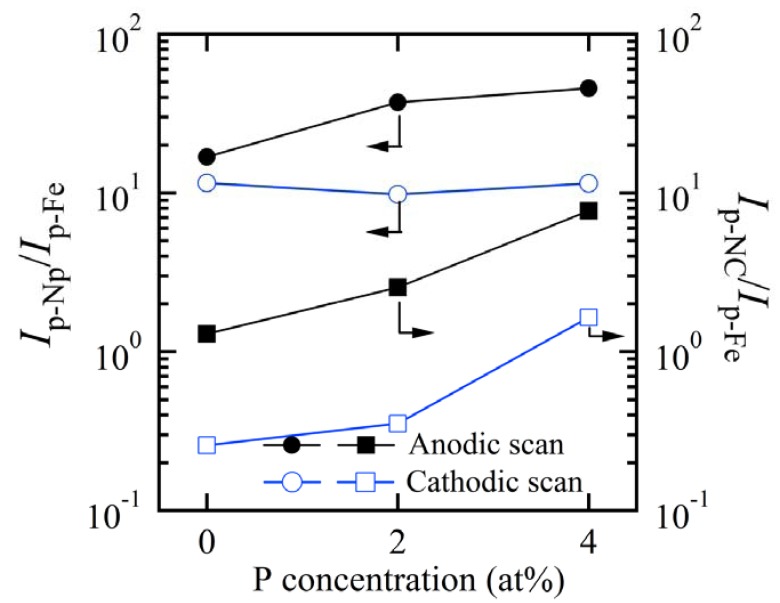
The increasing efficiency of Redox reaction of nanoporous and nanocrystalline Fe_85.2_B_14_Cu_0.8_, Fe_85.2_B_12_P_2_Cu_0.8_ and Fe_85.2_B_10_P_4_Cu_0.8_ electrodes with the P concentration normalized by the peak current density of Fe plate electrodes in 6 M KOH solution. *I*_p-Np_/*I*_p-Fe_: Ratio of the peak current density between nanoporous electrodes and Fe plate electrode; *I*_p-NC_/*I*_p-Fe_: Ratio of the peak current density between nanocrystalline electrodes and Fe plate electrode.

The Redox performance of nanocrystalline and nanoporous Fe-based alloys in 6.0 M KOH solution at room temperature is shown in Figure 8. The CV of Fe electrode in Figure 8c shows three current peaks at −1.09 V, −0.71 V and −0.50 V in the anodic scan and two peaks at −1.10 V and −1.23 V in the cathodic scan. As has been reported, the anodic peaks at −0.70 V and cathodic peak at −1.10 V correspond to the formation of FeOOH and the reduction of Fe^2+^ [30,31,32]. The CV curves of Fe_85.2_B_14_Cu_0.8_ and Fe_85.2_B_12_P_2_Cu_0.8_ precursors present small oxidization and reduction peaks in Figure 8a. The peak current density of nanocrystalline Fe_85.2_B_10_P_4_Cu_0.8_ precursor at −0.63 V was about 12 mA cm^−2^, about 4 times higher than those of pure Fe, Fe_85.2_B_14_Cu_0.8_ and Fe_85.2_B_12_P_2_Cu_0.8_ precursors. This might be due to the higher distribution of smaller α-Fe nanocrystals in the reaction surface of Fe_85.2_B_10_P_4_Cu_0.8_ precursor. The shape of the CV curves of nanoporous electrodes was inconsistent with the nanocrystalline alloys in Figure 8a. Those obtained from electrodes dealloyed from Fe-B-P-Cu precursors with a higher P concentration have the higher Redox peak current density in Figure 8b. The nanoporous electrode obtained from the Fe_85.2_B_14_Cu_0.8_ precursor consisted of single anodic current peaks at −0.72 V and one cathodic current peak at −1.15 V. The current density of the broad anodic peaks centering at −0.63 V for dealloyed Fe_85.2_B_10_P_4_Cu_0.8_ and −0.69 V for dealloyed Fe_85.2_B_12_P_2_Cu_0.8_ precursors was about 46 and 37 times higher than that of the flat Fe electrode, which indicates that the catalytic performance of the nanoporous electrode was superior in Redox properties. The enhanced Redox performance of the nanoporous electrodes in Figure 9 is reflected by the ratio of *I*_p-Np_/*I*_p-Fe_ and *I*_p-Nc_/*I*_p-Fe_ (the peak current densities of nanoporous, nanocrystalline electrodes after normalized by the peak current density of pure Fe electrodes at the corresponding potential). The ratio of nanoporous electrodes is higher than 10, and becomes larger with the increase of the P concentration. The enhancement of the Redox reactions on the nanocrystalline alloys is much weaker mainly because of the small active area of the nonporous alloys. In a word, the Redox performance is enhanced more than one order after the introduction of the nanoporous structure.

### 3.5. Discussion

As described above, the microstructure of amorphous alloys after flash annealing consisted of two phases: α-Fe nanocrystals and residual amorphous phase. As indicated by the polarization curves in Figure 4, the α-Fe nanocrystals acted as anodes and the adjacent amorphous region functioned as a cathode. The micro-coupling effect between α-Fe nanocrystals and the adjacent amorphous phases triggers the preferential dissolution of α-Fe nanocrystals [29]. The continuous distribution of the residual amorphous phase makes it possible to form the bi-continuous pore-and-ligament structure after preferential dissolution of α-Fe nanocrystals. This is the first trial to fabricate the nearly-amorphous nanoporous materials by dealloying the nanocrystalline precursor alloys. Moreover, the structural inheritance is determined by the unique dissolution mechanism of the nanocrystalline alloys. The formation process of the nanopores only includes two steps: the dissolution of α-Fe nanocrystals and the penetration of the dissolution front in the amorphous residue. This process has no rearrangement of noble adatoms, which differs from the typical dealloying process [2,6,32].

The enhanced Redox performance of the nanoporous electrodes is considered to be due to the large surface area and the nature of the nearly-amorphous ligaments. It is no doubt that the large surface area of the nanoporous materials certainly improves the electrochemical reactions [3,5,32]. The nanoporous electrodes mainly composed of amorphous residual phase have a Redox peak current density more than one order higher than those of pure iron and nanocrystalline electrodes in alkaline condition (Figure 8 and Figure 9). Wang [33] reported that higher energy state of amorphous structure endowed the electrons of surface atoms with lower activation energy to transfer. The surface of amorphous alloy has more unsaturated coordination, which can provide more active sites for chemical reactions. With theoretical calculations, Hu [34] pointed out that the bonding mechanism of atoms in amorphous structure was different from the case in crystalline structure. The excellent catalytic activity of amorphous electrodes resulted from the abundance of the active sites induced by the special electronic structure. Therefore, the nanoporous electrodes mainly composed of amorphous residual phase demonstrated much better Redox performance than pure Fe and nanocrystalline electrodes. This strategy can be extended to other nanocrystalline alloys with two or more phases with different electrochemical stabilities for the fabrication of porous materials.

## 4. Conclusions

A nanoporous Fe-based nearly-amorphous material with uniformly distributed nanoscale porosity was fabricated on nanocrystalline precursor alloys. The nanopores formed at the sites where α-Fe nanocrystals distributed and the nanopores were similar in size to the particle size of α-Fe nanocrystals. The nanoporous structure inherits the characteristics of the nanocrystalline precursor alloys. It is possible to predict the final nanoporosity of the nanoporous nearly-amorphous materials from the microstructure of the nanocrystalline precursor alloys. The microstructure of the nanocrystalline alloys became more uniform and the α-Fe nanocrystals became smaller with increasing P content. With these findings in mind, it is easy to envision the refinement of nanoporous structures on other P containing precursor alloys. The peak current density of nanoporous electrodes mainly composed of amorphous phases are more than one order higher than flat Fe and nanocrystalline electrodes due to their large surface areas and the nearly-amorphous nature of the ligaments.

## Supplementary Materials

The following are available online at http://www.mdpi.com/2079-4991/7/6/141/s1.

## References

[1] Zhang Z.H., Wang Y., Qi Z., Zhang W.H., Qin J.Y., Frenzel J. (2009). Generalized Fabrication of Nanoporous Metals (Au, Pd, Pt, Ag, and Cu) through Chemical Dealloying. J. Phys. Chem. C.

[2] Erlebacher J., Aziz M.J., Karma A., Dimitrov N., Sieradzki K. (2001). Evolution of Nanoporosity in Dealloying. Nature.

[3] Bond G.C., Thompson D.T. (1999). Catalysis by Gold. Catal. Rev..

[4] Weissmueller J.R., Viswanath N., Kramer D., Zimmer P., Wuerschum R., Gleiter H. (2003). Charge-induced Reversible Strain in a Metal. Science.

[5] Joo S.H., Choi S.J., Kwa K.J., Liu Z., Terasaki O., Ryoo R. (2001). Ordered Nanoporous Arrays of Carbon Supporting High Dispersions of Platinum Nanoparticles. Nature.

[6] Erlebacher J. (2004). An Atomistic Description of Dealloying: Porosity Evolution, the Critical Potential, and Rate-limiting Behavior. J. Electrochem. Soc..

[7] Jayaraj J., Park B.J., Kim D.H., Kim W.T., Fleury E. (2006). Nanometer-sized Porous Ti-based Metallic Glass. Scr. Mater..

[8] Gebert A., Kündig A.A., Schultz L., Hono K. (2004). Selective Electrochemical Dissolution in Two-phase La–Zr–Al–Cu–Ni Metallic Glass. Scr. Mater..

[9] Dan Z.H., Qin F.X., Sugawara Y., Muto I., Hara N. (2013). Dealloying Behaviours of an Equiatomic TiCu Alloy. Mater. Trans..

[10] Tsuchiya H., Akaki T., Koizumi Y., Minamino Y., Fujimoto S. (2013). Selective Pore Growth on Lamellar Ti–41at %Al Alloy. Electrochem. Commun..

[11] Mihailov L., Redzheb M., Spassov T. (2013). Selective Dissolution of Amorphous and Nanocrystalline Zr_2_Ni. Corros. Sci..

[12] Rösler J., Näth O., Jäger S., Schmitz F., Mukherji D. (2005). Fabrication of Nanoporous Ni-based SuperalloyMembranes. Acta Mater..

[13] Makino A., Men H., Kubota T., Yubuta K., Makabe M., Inoue A. (2009). New Excellent Soft Magnetic FeSiBPCu Nanocrystallized Alloys with High B_s_ of 1.9 T from Nanohetero-Amorphous Phase. IEEE Trans. Magn..

[14] Makino A., Kubota T., Yubuta K., Inoue A., Urata A., Matsumoto H., Yoshida S. (2011). Low Core Losses and Magnetic Properties of Fe_85–86_Si_1–2_B_8_P_4_Cu_1_Nanocrystalline Alloys with High B for Power Applications. J. Appl. Phys..

[15] Makino A., Men H., Yubuta K., Kubota T. (2009). New Fe-metalloids Based Nanocrystalline Alloys with High B_s_ of 1.9 T and Excellent Magnetic Softness. J. Appl. Phys..

[16] Lee M.H., Sordelet D.J. (2006). Nanoporous Metallic Glass with High Surface Area. Scr. Mater..

[17] Li X., Kato H., Yubuta K., Makino A., Inoue A. (2010). Effect of Cu on nanocrystallization and plastic properties of FeSiBPCu bulk metallic glasses. Mater. Sci. Eng. A.

[18] Sharma P., Zhang X., Zhang Y., Makino A. (2015). Competition Driven Nanocrystallization in High B_s_ and Low Coreloss Fe-Si-B-P-Cu Soft Magnetic Alloys. Scr. Mater..

[19] Makino A., Kubota T., Chang C.T., Makabe M., Inoue A. (2008). FeSiBP Bulk Metallic Glasses with High Magnetization and Excellent Magnetic Softness. J. Magn. Magn. Mater..

[20] Dan Z.H., Makino A., Hara N. (2013). Effects of P Addition on Corrosion Properties of Soft Magnetic FeSiB Alloys. Mater. Trans..

[21] Leofantia G., Padovanb M., Tozzolac G., Venturelli B. (1998). Surface area and pore texture of catalysts. Catal. Today.

[22] Wang Z.L., Xu D., Xu J.J., Zhang X.B. (2014). Oxygen Electrocatalysts in Metal–air Batteries: From Aqueous to Nonaqueous Electrolytes. Chem. Soc. Rev..

[23] Manohar A.K., Malkhandi S., Yang B., Yang C.G., Prakash G.S.K., Narayanan S.R. (2012). High-performance rechargeable iron electrode for large-scale battery-based energy storage. J. Electrochem. Soc..

[24] Ojefors L., Carlsson L. (1978). An Iron-air Vehicle Battery. J. Power Sources.

[25] Cullity B.D., Stock R.S. (1978). Elements of X-ray Diffractions.

[26] Inoue A. (2000). Stabilization of Metallic Supercooled Liquid and Bulk Amorphous Alloys. Acta Mater..

[27] Brett C.M.A., Brett A.M.O. (1998). Electrochemistry: Principles, Methods and Application.

[28] Pickering H.W. (1983). Characteristic Features of Alloy Polarization Curves. Corros. Sci..

[29] Dan Z.H., Qin F.X., Zhang Y., Makino A., Chang H., Hara N. (2016). Mechanism of Active Dissolution of Nanocrystalline Fe-Si-B-P-Cu Soft Magnetic Alloys. Mater. Charact..

[30] Ojefors L. (1976). Self-discharge of the Alkaline Iron Electrode. Electrochim. Acta.

[31] Beck F., Kaus R., Oberst M. (1985). Transpassive Dissolution of Iron to Ferrate (VI) in Concentrated Alkali Hydroxide Solutions. Electrochim. Acta.

[32] Synder J., Livi K., Erlebacher J. (2008). Dealloying Silver/gold Alloys in Neutral Silver Nitrate Solution: Porosity Evolution, Surface Composition, and Surface Oxides. J. Electrochem. Soc..

[33] Wang J.Q., Liu Y.H., Chen M.W., Xie G.Q., Louzguine-Luzgin D.V., Inoue A., Perepezko J.H. (2012). Rapid Degradation of Azo dye by Fe-based Metallic Glass Powder. Adv. Funct. Mater..

[34] Hu Y.C., Wang Y.Z., Su R., Cao C.R., Li F., Sun C.W., Yang Y., Guan P.F., Ding D.W., Wang Z.L. (2016). A Highly Efficient and Self-stabilizing Metallic-glass Catalyst for Electrochemical Hydrogen Generation. Adv. Mater..

