# Supplementary material for: Structural Inheritance and Redox Performance of Nanoporous Electrodes from Nanocrystalline Fe85.2B10-14P0-4Cu0.8 Alloys
Source: Nanomaterials (Basel). 2017 Jun 8;7(6):141. doi: 10.3390/nano7060141 (PMC5485788; doi:10.3390/nano7060141)
Supplement: Supplementary file 1 [file nanomaterials-07-00141-s001.pdf]

Supplementary figure:

Fig. S1 shows the elemental mapping of the nanocrystalline $Fe_{85.2}B_{10}P_4Cu_{0.8}$ alloys at the region where the one α-Fe nanocrystal distributed. The seed element, Cu is randomly distributed. The P element is enriched in the adjacent amorphous phase, and on the other hand Fe element has a higher concentration in the crystalline phase (α-Fe nanocrystals).

Fig. S2 presents the collected data of the particle size and pore size the grain size of a-Fe nanocrystals (a, b) in as-annealed $Fe_{85.2}B_{14}Cu_{0.8}$ and $Fe_{85.2}B_{10}P_4Cu_{0.8}$ alloys and pore size (c, d) in as-dealloyed $Fe_{85.2}B_{14}Cu_{0.8}$ and $Fe_{85.2}B_{10}P_4Cu_{0.8}$ alloys. The value of D_2 and d_2 was obtained from the three TEM images similar with Figs. 2a, d and 4a, d for each condition. The data of D_2 and d_2 were obtained from more than 125 sites for each TEM image by using Nanomeasure® software. The distribution ratio of α-Fe nanocrystals in annealed $Fe_{85.2}B_{14}Cu_{0.8}$ alloys in Fig. S2 b and nanopores in dealloyed $Fe_{85.2}B_{10}P_4Cu_{0.8}$ alloys in Fig. S2 d is typical of the normal distribution. On the other hand, that of α-Fe nanocrystals in annealed $Fe_{85.2}B_{10}P_4Cu_{0.8}$ alloys in Fig. S2 a and nanopores in dealloyed $Fe_{85.2}B_{14}Cu_{0.8}$ alloys in Fig. S2 c shows a larger divergence, particular the α-Fe nanocrystals with a large size of more than 60 nm and nanopores with a pore size of larger than 30 nm. The data demonstrate that the uniformity of the nanoporous structure in dealloyed $Fe_{85.2}B_{10}P_4Cu_{0.8}$ alloys is better than that of dealloyed $Fe_{85.2}B_{10}P_4Cu_{0.8}$ alloys due to the worse microstructure of annealed $Fe_{85.2}B_{14}Cu_{0.8}$ alloys with irregular-shaped a-Fe particles.

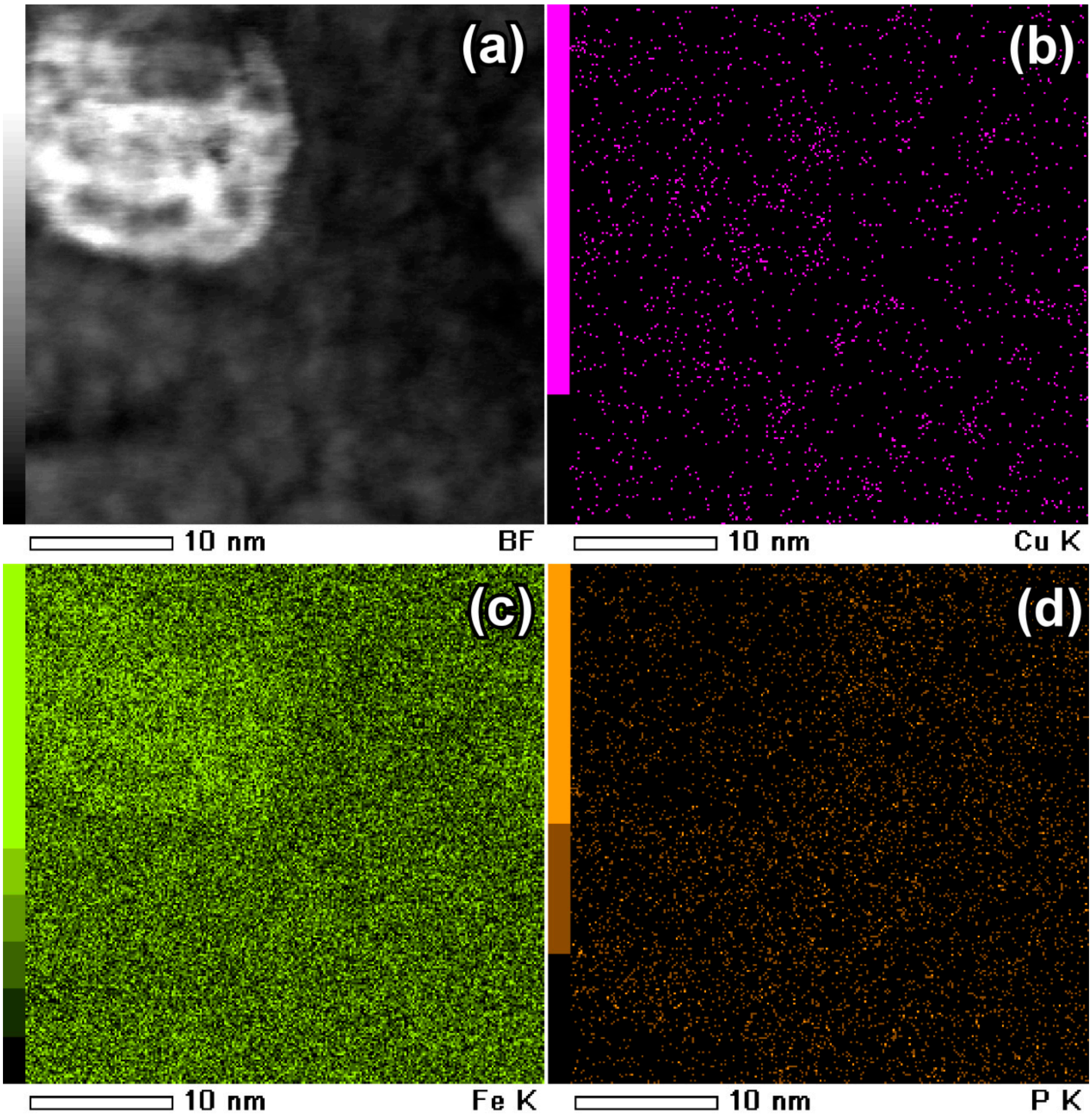

Fig. S1 BFI TEM image (a) and elemental mapping of Cu (b), Fe(c) and P (d) elements in the as-annealed $Fe_{85.2}B_{10}P_4Cu_{0.8}$ alloys.

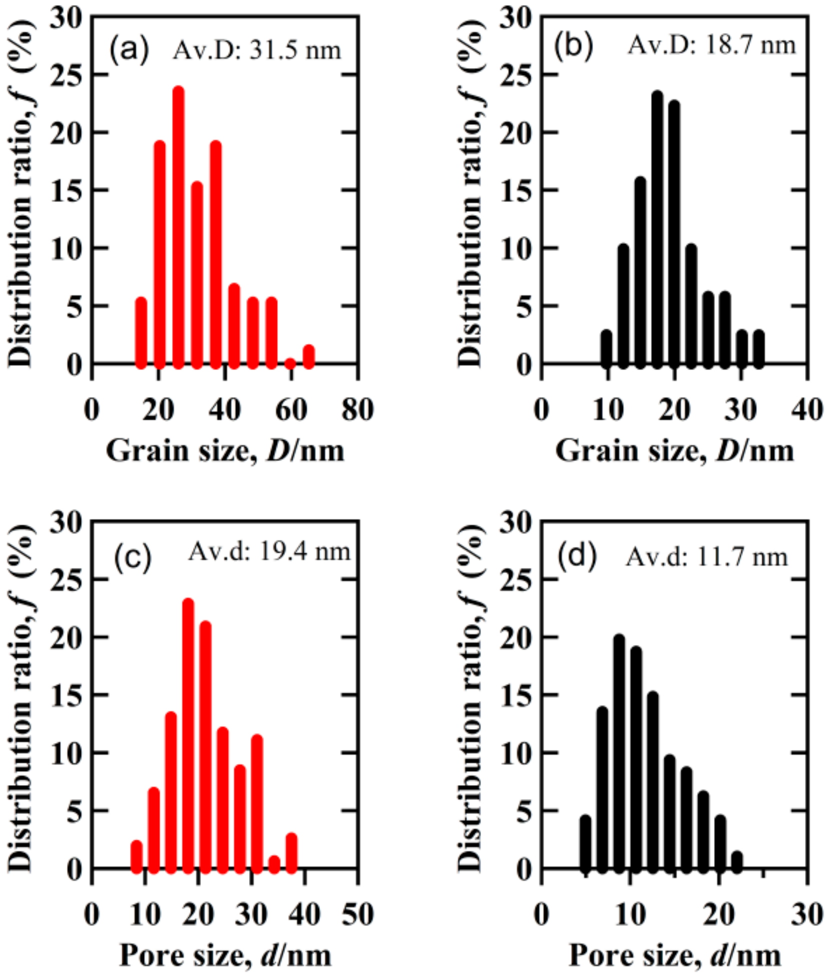

Fig. S2 The distribution ratio of the grain size of a-Fe nanocrystals (a, b) in as-annealed $Fe_{85.2}B_{14}Cu_{0.8}$ and $Fe_{85.2}B_{10}P_4Cu_{0.8}$ alloys and pore size (c, d) in as-dealloyed $Fe_{85.2}B_{14}Cu_{0.8}$ and $Fe_{85.2}B_{10}P_4Cu_{0.8}$ alloys.
